# Proteins journey—from marine to freshwater ecosystem: blood plasma proteomic profiles of pink salmon *Oncorhynchus gorbuscha* Walbaum, 1792 during spawning migration

**DOI:** 10.3389/fphys.2023.1216119

**Published:** 2023-06-13

**Authors:** Albina Kochneva, Denis Efremov, Svetlana A. Murzina

**Affiliations:** ^1^ Environmental Biochemistry Laboratory, Institute of Biology of the Karelian Research Centre of the Russian Academy of Sciences, Petrozavodsk, Russia; ^2^ Ecology of Fishes and Water Invertebrates Laboratory, Institute of Biology of the Karelian Research Centre of the Russian Academy of Sciences, Petrozavodsk, Russia

**Keywords:** *Oncorhynchus gorbuscha*, pink salmon, proteomics, blood plasma, anadromous migration, spawning

## Abstract

The pink salmon (*Oncorhynchus gorbuscha*) is a commercial anadromous fish species of the family Salmonidae. This species has a 2-year life cycle that distinguishes it from other salmonids. It includes the spawning migration from marine to freshwater environments, accompanied by significant physiological and biochemical adaptive changes in the body. This study reveals and describes variability in the blood plasma proteomes of female and male pink salmon collected from three biotopes—marine, estuarine and riverine—that the fish pass through in spawning migration. Identification and comparative analysis of blood plasma protein profiles were performed using proteomics and bioinformatic approaches. The blood proteomes of female and male spawners collected from different biotopes were qualitatively and quantitatively distinguished. Females differed primarily in proteins associated with reproductive system development (certain vitellogenin and choriogenin), lipid transport (fatty acid binding protein) and energy production (fructose 1,6-bisphosphatase), and males in proteins involved in blood coagulation (fibrinogen), immune response (lectins) and reproductive processes (vitellogenin). Differentially expressed sex-specific proteins were implicated in proteolysis (aminopeptidases), platelet activation (β- and γ-chain fibrinogen), cell growth and differentiation (a protein containing the TGF_BETA_2 domain) and lipid transport processes (vitellogenin and apolipoprotein). The results are of both fundamental and practical importance, adding to existing knowledge of the biochemical adaptations to spawning of pink salmon, a representative of economically important migratory fish species.

## 1 Introduction

The pink salmon (*Oncorhynchus gorbuscha*, Walbaum, 1792) belongs to the Salmonidae family and has an anadromous life strategy: a few weeks before the final maturation of the gonads, adult fish migrate from seawater to a freshwater spawning ground for spawning and further breeding (Gallagher et al., 2012; Morita, 2022). Pink salmon usually have a strict 2-year life cycle (Paulsen et al., 2022). The gene flow between annual lines of pink salmon is limited; this phenomenon is known as allochronic or temporal isolation (Aspinwall, 1974). Genetic differentiation between odd and even lineages from the same river is greater than differentiation by year, a phenomenon observed throughout the species’ natural range (Tarpey et al., 2018). In August–September, after spawning in a river, pink salmon spawners die, and in November–January, larvae hatch and remain in and then close to the spawning nest until spring. During the winter, the larvae feed from the yolk sac is fully absorbed by spring. In May–April, the young of *Oncorhynchus gorbuscha* have transformed into saline-tolerant smolts that migrate downstream to estuaries and coastal waters, where they remain for several months for feeding (Heard, 1991; Quinn, 2005; Gallagher et al., 2012; Paulsen et al., 2022). Notably among salmonids, the pink salmon has no parr stage in ontogeny. Pink salmon at the 1+ age return to freshwater ecosystems—rivers—to spawn after a year and a half at sea (Christensen et al., 2021).

The physiological profile of pink salmon spawning is characterized by a number of features. Spawning adults stop feeding and lose approximately 50% of their body weight to compensate for starvation and locomotor function, gonad formation, hypoosmotic adaptation, aggressive behaviour, physical activity limitation, and condition of the fish (Cook et al., 2011; Nemova et al., 2021). During spawning, there are significant increases in contents of cortisol, lactate, and glucose in the blood plasma of fish (Cooke et al., 2006; Crossin et al., 2009), which ensures the processes of homing (Carruth et al., 2002), accelerates ovulation (Schreck, 2010), but at the same time leads to a weakening of the immune system (Maule et al., 1996) and irreversible degenerative processes in tissues and organs (Hendry and Berg, 1999; Maldonado et al., 2000). The post-spawning death of pink salmon, a semelparous species, is largely due to an excessive concentration of cortisol in the blood plasma (Cook et al., 2011).

The migration of pink salmon is accompanied by a number of biochemical and physiological adaptations, the cumulative effect of which lead to a successful switch from a marine to a freshwater ecosystem for final spawning (Hinch et al., 2005; Wilson et al., 2014). Previous studies have demonstrated changes in metabolism. For example, . For example, indicators of oxidative stress (including DNA damage) in pink salmon females increase in heart tissues and blood plasma after entry into river (Wilson et al., 2014); tissue-specific fatty acid profiles switch from a “marine” to a “freshwater” type of lifestyle (Murzina et al., 2018); thyroid and sex steroid hormone changes in spawners. Compared with individuals in the sea, females in river are characterized by an increased level of triiodothyronine and an increased rate of deiodination, while males have an increased level of testosterone. Females both in the sea and in river differ from males by a higher content of estradiol-17β and a lower ratio of testosterone to estradiol-17β concentrations (Pavlov et al., 2022).

An essential role in the molecular processes of vital body activity is performed by multifunctional proteins. The blood composition of fish can include various groups of proteins (immunoglobulins, transport proteins, intracellular proteins that enter the plasma when cells are destroyed or have increased permeability, etc.), so haematological parameters can serve as markers of changes in the state of fish or any other living system, especially poikilotherms, when changing environment. Proteomics approaches are used to identify and to qualitatively and quantitatively analyse protein patterns in cells and tissues, which makes it possible to describe the current state of the body’s metabolism, including when exposed to various factors of internal and external environments.

There are few studies (Miller et al., 2009; Kanerva et al., 2014; Morro et al., 2020) on protein change and the importance of certain shifts during migration of salmonids discussing the biological importance and implementation of such findings in biodiversity and conservation programmes and in Salmonid breeding approaches. In the present study, qualitative and quantitative changes in the blood plasma proteome compositions of female and male pink salmon *O. gorbuscha* collected from three biotopes—marine, estuarine and riverine—consequentially passed by spawners during spawning migration are evaluated and discussed. The results are of fundamental importance and have practical applications as they develop existing knowledge about biochemical adaptations during the spawning migration of pink salmon, which is a representative of commercial anadromous fish species.

## 2 Materials and methods

### 2.1 Sample collection

Pink salmon *O. gorbuscha* (males and females) were caught during the pre-spawning period (August 10–15, 2021) at three biotopes in the White Sea—“marine” (66°14'12.0"N 37°08'58.8"E), the estuaries of the Indera River—“estuarine” (66°14'28.6"N 37°08'55.8"E) and directly in the Indera River—“freshwater” (66°14'34.6"N 37°08'55.8"E). The fish was caught using a casting net (mesh 15–20 mm) and gillnet (mesh 55 mm). The water temperature in the river during fishing was +16.3°С, in the sea +19.2°С, and in the estuary +16.8°С. The fish were collected from cages and transferred to eurocubes (5 individuals each) with salt water, freshwater and a mixture of fresh and salt water (1:1, v:v), where they were kept for at least 2 h (no more than 10–11 h). The water in the cubes was aerated using a compressor. Its temperature varied between 17°C and 19°C depending on the time of day. Fish were quickly dip-netted out of the cubes and were treated of clove oil for blood collection. Blood was extracted using syringes immediately when fish lost equilibrium and did not move. The blood samples were centrifuged at 1700 g for 10 min to obtain plasma, which was frozen and stored in liquid nitrogen.

For each fish, weight, length and maturity stage were recorded (Table 1). Permit for pink salmon producers collection No. 51 2021 03 2021 were issued by the North Sea Territorial Administration of the Federal Fisheries Agency on 06/19/2021.

**TABLE 1:** Description of the analyzed pink salmon individuals.

No fish	Biotope	Sex	Weight, kg	Length AB, cm	Maturity stage
7	Sea	Male	0.740	45.5	4–5
11	Sea	Male	0.770	44.5	4–5
13	Sea	Male	0.840	46.0	4–5
9	Sea	Female	0.995	46.0	4
10	Sea	Female	0.875	44.5	4
12	Sea	Female	0.790	45.0	4–5
14	Estuary	Male	1.460	52.5	4
15	Estuary	Male	0.925	48.0	4–5
17	Estuary	Male	1.670	52.5	4–5
8	Estuary	Female	1.030	46.5	5
16	Estuary	Female	1.020	48.0	4–5
18	Estuary	Female	0.980	46.5	4–5
3	River	Male	1.230	53.0	4
4	River	Male	1.405	52.0	4
5	River	Male	0.995	49.5	4
1	River	Female	1.147	48.0	4
2	River	Female	1.065	46.5	4
6	River	Female	1.055	46.5	4

### 2.2 Proteomic analysis

#### 2.2.1 Sample preparation

The samples were thawed, 100 µL were taken, and centrifuged at 20,000 g for 10 min. The supernatant was used for further analysis. The protein concentration in the samples was measured by the BCA method. In 1.5 mL tubes were mixed: 3 µL of sample (supernatant), 20 µL of water, 1 mL of BCA reagent (0.2 g of BCA, 4 g of sodium carbonate, 0.32 g of sodium tartrate, 0.8 g of sodium hydroxide, 1.9 g of sodium bicarbonate), 20 µL of 4% copper sulfate solution. A control sample was prepared by mixing: 30 µL of water, 1 mL of BCA reagent, 20 µL of 4% copper sulfate solution. Similarly, a calibration curve was constructed (solutions of bovine serum albumin at a concentration (10 mg/mL (Gerbu, #1062) from 0.0667 μg/μL to 2.5 μg/μL) with the BCA reagent. The samples were shaken and incubated for 20 min at 56°C on a Termomixer thermal shaker (Eppendorf, Germany) with stirring. The samples were analyzed on a Clarion instrument (BMG LAbtech, Germany) at a wavelength of 562 nm in three replicates after cooling to room temperature. A sample amount equivalent to 100 μg of protein was taken for analysis.

Sample preparation was carried out according to the S-trap protocol (HaileMariam et al., 2018). 10% SDS in 100 mM TEAB was added to the samples to give a final SDS concentration of 5% (sample lysis buffer: 10% SDS in 100 mM TEAB). Mixed and centrifuged at high speed to precipitate the foam. 2 μL of 0.5 M TCEP (Supelco, #646547) was added to the samples, shaken, and rapidly centrifuged; 4 μL of 400 mM chloroacetamide (Acros, #148410010) in 50 mM TEAB was added. Vortex, centrifuge and incubate at 80°C for 30 min. The samples were cooled to room temperature. After the reduction and alkylation, a 12% phosphoric acid solution was added to the samples in a volume equal to 10% of the sample volume. Final acid concentration of 1%, pH of the sample is less than 1. A 6-fold volume of 90% MeOH buffer in 100 mM TEAB was then added and mixed by pipetting. 170 μL of the samples were applied to a Strap filter (ProtiFi LLC) and centrifuged at 4,000 g for 3 min. To wash the samples from SDS, 170 μL of 90% MeOH in 100 mM TEAB was applied to the filters, centrifuged at 4,000 g for 3 min, and this step was repeated 3 more times.

The filters were transferred to clean 1.5 mL tubes, 100 µL of 50 mM TEAB and 1 µg of trypsin (Promega, #V5111) (trypsin:protein ratio 1:100) were added and incubated for 1 h at 37°C. Then 1 µg trypsin was added again (final trypsin:protein ratio 1:50) and incubated for 18 h (overnight) at 37°C. To elute the peptides, 80 µL of 50 mM TEAB was added to the filters, centrifuged at 4,000 g for 3 min. Then 80 µL of 0.2% formic acid in water was added and centrifuged at 4,000 g for 3 min, 80 µL of 50% acetonitrile in water with 0.1% formic acid was added and centrifuged at 4,000 g for 3 min. The washes obtained were combined in a glass insert for the HPLC-MS analysis, evaporated in a vacuum concentrator, and redissolved in 20 µL of a 0.1% aqueous formic acid solution.

#### 2.2.2 Chromatography mass-spectrometric analysis

Chromatography-mass spectrometry analysis of the samples after hydrolysis was performed using an Ultimate 3,000 RSLCnano chromatographic HPLC system coupled to a Q-Exactive HF-X mass spectrometer.

Samples of 3 µL were applied to an Acclaim µ-Precolumn enrichment column (0.5 mm × 3 mm, 5 µm particle size, Thermo Scientific) at a flow rate of 10 μL/min for 5 min in isocratic mode using buffer “C” as the mobile phase (2% acetonitrile, 0.1% formic acid in deionized water). The peptides were separated on an Acclaim Pepmap^®^ C18 HPLC column (75 μm × 150 mm, 2 µm particle size) (Thermo Scientific, United States) in gradient elution mode. The gradient was formed by mobile phase A (0.1% formic acid in deionized water) and mobile phase B: (80% acetonitrile, 0.1% formic acid in deionized water). The eluent flow rate was 400 nL/min. The column was washed with 2% mobile phase B for 1 min, the mobile phase B concentration was increased linearly over 4 min to 10%, then the concentration of mobile phase B was increased linearly over 90 min to 40%, then the concentration of mobile phase B was increased linearly over 3 min to 99%, after a 5 min wash with 99% mobile phase B, the concentration of this buffer was reduced linearly over 3 min to the original 2% and the column was washed with 2% mobile phase B for 5 min. The total analysis time was 110 min.

Mass spectrometric analysis was performed on a Q Exactive HF-X mass spectrometer in positive ionization mode using a NESI source (Thermo Scientific). The following parameters were set: emitter voltage 2.1 kV, capillary temperature 240°C. Panoramic scans were performed in the mass range from 390 m/z to 1,400 m/z with a 120,000 resolution. Tandem scans were performed in the mass range from 140 m/z to the upper limit automatically determined from the precursor mass, but not exceeding 2000 m/z, at 15,000 resolution. The precursor ions were isolated within a window of ± 2 Da. The maximum number of ions allowed for isolation in MS/MS mode was 20 or less, the minimum precursor ion intensity for tandem analysis was set at 50,000 units, and the normalized impact energy was 29. Only ions from z = 2+ to z = 4+ by charge state were considered for tandem scanning. The maximum accumulation time was 50 ms for precursor ions and 70 ms for fragment ions. All measured precursors were dynamically excluded from the tandem MS/MS analysis for 9 s. Samples were analyzed in three replicates.

### 2.3 Bioinformatics and statistical data processing

The mass spectra raw-files were loaded into the MaxQuant v.1.6.15.0 program with the built-in Andromeda search algorithm (Cox et al., 2011; Tyanova et al., 2016а). The search was performed using Oncorhynchus spp. database provided by UniProt (June 2022). The following search parameters were set: the degrading enzyme—trypsin; accuracy of monoisotopic peptide masses ±5ppm; the accuracy of the masses in the MS/MS spectra ± 0.01 Da; the possibility of skipping two sites of trypsin cleavage; methionine oxidation, N-terminal acetylation and cysteine modification with iodoacetamide were set as variable and fixed modification of peptides, respectively; for validation of comparisons (pairing) of spectra and PSM peptides (Peptide Spectrum Matches), identification of peptides and proteins, the FDR value (False Discovery Rate) no more than 1.0%. Proteins were considered to be reliably identified if at least two peptides were detected for them. The quantification of protein content was based on the label-free quantification (LFQ) algorithm.

Statistical analysis of the obtained data was performed in Perseus v.1.6.15.0 software (Tyanova et al., 2016b). The data were pre-filtered to select the most significant points. Possible contaminating proteins, proteins identified by modified and reversed peptides, proteins with less than two unique peptides, and proteins that were present in less than 70% of the female and male group samples from each biotope analyzed were removed. Then, the LFQ intensity values of the spectra are logarithmized and the missing values in the sample are imputed. Comparative quantitative analysis of pink salmon protein profiles was performed using the Student’s t-test (*p*-value ≤ 0.05) and permutation-based FDR truncation (q-value ≤ 0.01). The complete absence of MS/MS peaks in all technical and biological replicates of the samples in the analyzed groups was defined as a qualitative difference. R and RStudio software (R Core Team, 2016; RStudio Team, 2020) was used for cluster analysis and visualization data using the “readxl” (Wickham and Bryan, 2023), “factoextra” (Kassambara and Mundt, 2020), “ComplexHeatmap” (Gu et a., 2016), “ggplot2” (Wickham, 2016), “cowplot” (Wilke, 2020), “gridExtra” (Auguie, 2017), “ggpubr” (Kassambara., 2023). Functional annotation of the identified proteins was performed using the InterPro and QuickGO services (Binns et al., 2009; Paysan-Lafosse et al., 2023).

The mass spectrometry proteomics data have been deposited to the ProteomeXchange Consortium (http://proteomecentral.proteomexchange.org) via the PRIDE partner repository (Vizcaíno et al., 2013) with the dataset identifier <PXD042278>.

## 3 Results

### 3.1 Cluster analysis proteomic profiles of *O. gorbuscha* blood plasma

**FIGURE 1:**
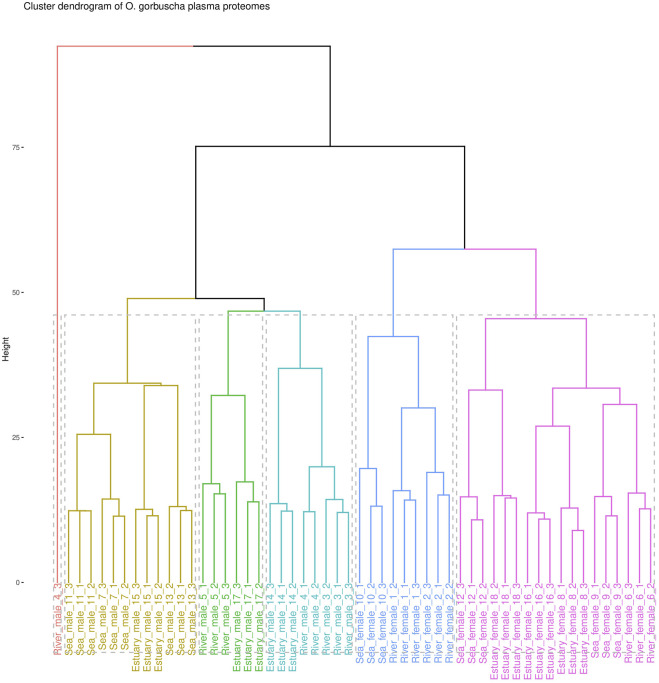
Cluster analysis dendrogram of blood plasma proteomic profiles of *O. gorbuscha* males and females from different biotopes (marine, estuarine and riverine). Note: The color shows that the sample belongs to one of six clusters.

The proteomic analysis identified a total of 716 proteins, among which 412 were selected for comparative analysis after filtering and missing values imputation (Supplementary Tables S1–S3). To assess the homogeneity of the analysed data, a cluster analysis was performed (Figure 1). Data clearly defines two groups according to sex. However, there is some heterogeneity within the female and male groups due to the overlap of samples from the studied biotopes. The males from the “marine” and “riverine” biotopes formed two separate clusters, while males from the estuary overlapped with others. It was observed that females collected from each biotope were characterized by greater heterogeneity than males. Females (except for one sample) collected from the river or from the estuary indicated two separate clusters, and fish from the marine biotope overlapped with those from the riverine and estuary.

### 3.2 Comparative analysis of plasma protein profiles of *O. gorbuscha* females from different biotopes—marine, estuarine and riverine

**FIGURE 2:**
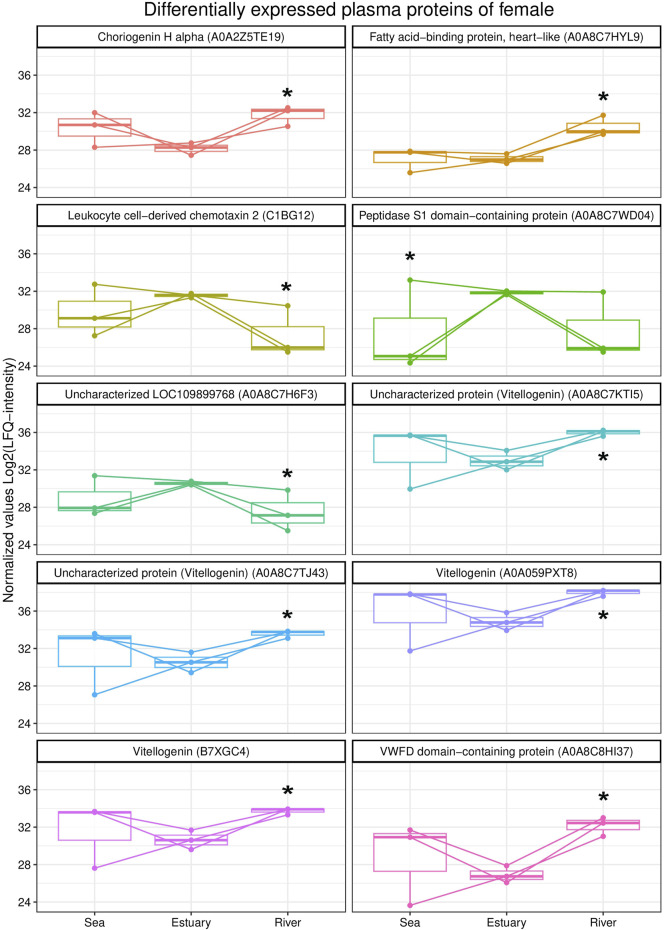
Box plots of differentially expressed blood plasma proteins of female *O. gorbuscha* from different biotopes (marine, estuarine and riverine). * - Differences are statistically significant compared to females from estuary at *p*-value ≤ 0.05 and q-value ≤ 0.01. The size of each sample is (sea, estuary, river) n = 3.

A comparative analysis of the blood plasma protein profiles of females revealed qualitative and statistically significant quantitative differences (Figure 2). Females from the river were characterized by higher levels of three vitellogenin isoforms, namely, vitellogenin As, uncharacterized proteins (A0A8C7TJ43 and A0A8C7KTI5), vitellogenin, choriogenin H alpha, fatty acid binding protein,heart-like (FABP), and von Willebrand factor type D (VWFD) domain-containing protein) compared to those from the estuary. Further, levels of uncharacterized protein LOC109899768, leukocyte chemotaxin 2, and peptidase S1 domain-containing protein were lower in females from either the riverine or marine biotope than in fish from the estuary.

Qualitative differences were found in the composition of plasma protein profiles of O*. gorbuscha* females from different biotopes. The coatomer subunit beta'-like, fructose bisphosphatase, FABP 10-A liver basic and 4-hydroxyphenylpyruvate dioxygenase were identified only in the blood plasma of females from the river.

### 3.3 Comparative analysis of plasma protein profiles of *O. gorbuscha* males from different biotopes—marine, estuarine and riverine

**FIGURE 3:**
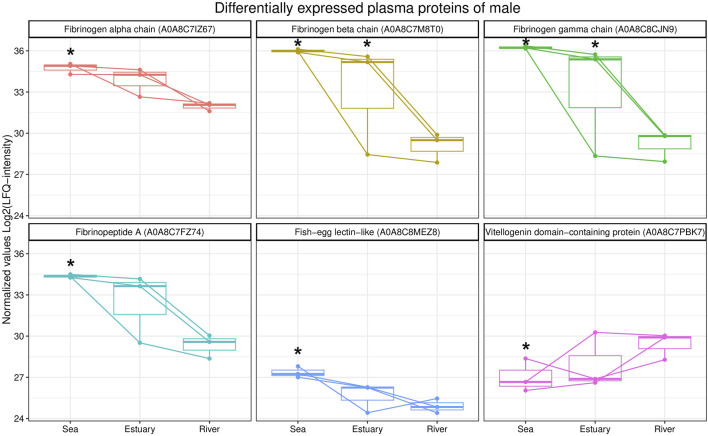
Box plots of differentially expressed blood plasma proteins from male *O. gorbuscha* from different biotopes (marine, estuarine and riverine). *—Differences are statistically significant compared to male from river at *p*-value ≤ 0.05 and q-value ≤ 0.01. The size of each sample is (sea, estuary, river) n = 3.

A comparative analysis of the blood plasma protein profiles of male pink salmon collected in different biotopes determined qualitative and statistically significant quantitative differences (Figure 3). During their transition from the sea to the river, the levels of several isoforms of fibrinogen (fibrinogen alpha, beta and gamma chains, fibrinopeptide A) and fish-egg lectin (FEL)-like protein decreased in the blood, whereas the level of protein containing the vitellogenin domain increased.

Notably, mannose-specific lectin-like (MBL) was identified only in males from the estuary and the river. In addition, the enzyme peptidyl-prolyl cis-trans isomerase (PPIase) was identified only in the plasma of males collected from the sea.

### 3.4 Comparative proteomic analysis of male and female *O. gorbuscha* plasma

**FIGURE 4:**
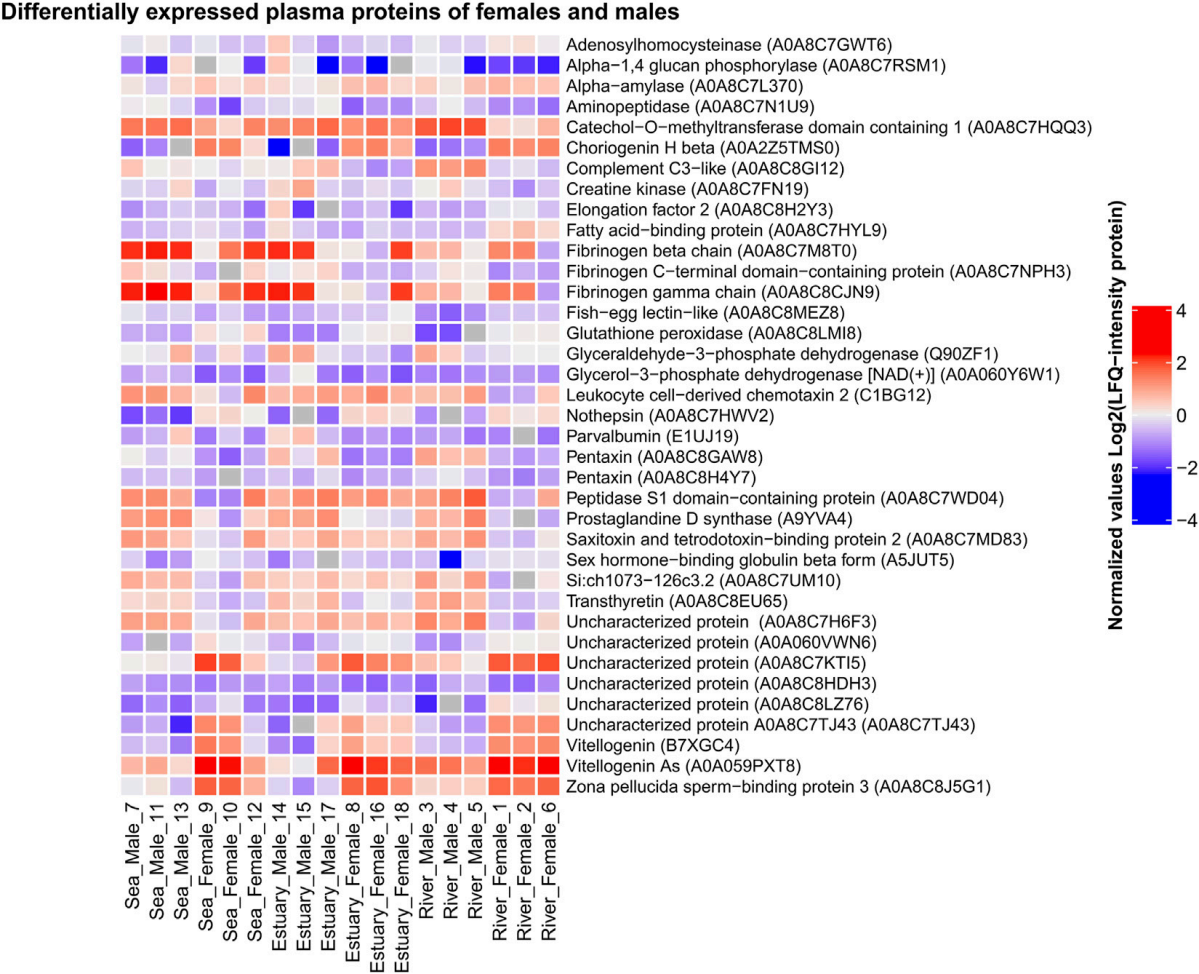
Heat map of differentially expressed plasma proteins of *O. gorbuscha* male and female from different biotopes (marine, estuarine and riverine). The color scale gradation performs the protein level in the sample from lowest (blue) to highest (red). Differences are statistically significant at *p*-value ≤ 0.05 and q-value ≤ 0.01. The size of each sample is (sea, estuary, river) n = 3.

Comparative analysis of the blood plasma protein composition of males and females collected from all three biotopes (consequentially passed during spawning migration) revealed statistically significant quantitative (Figure 4; Supplementary Table S4) and qualitative differences.

Females from all biotopes showed higher levels of multiple isoforms of vitellogenin, choriogenin, zona pellucida sperm-binding protein 3, and glutathione peroxidase, whereas males from all biotopes showed increased levels of aminopeptidase, fibrinogen C-terminal domain-containing protein, pentaxin, and prostaglandin D-synthase. There were also differences in blood plasma protein levels between males and females that were not observed in fish from all three habitats. For example, higher levels of sex hormone-binding globulin beta form and lower levels of catechol-O-methyltransferase domain containing 1 were observed in females compared to males only in the sea and river. More detailed information on the sex-specific variability of pink salmon plasma proteins during spawning is presented in the Supplementary Table S4.

Unique (sex-specific) proteins present only in one or the other group were identified by comparing the proteomic profiles of males and females in each biotope. Vitronectin b and protein containing the TGF_BETA_2 domain were identified only in the males’ blood plasma from sea and estuary, and ceramidase, an uncharacterized protein A0A060Z7F0, and a CD59-like -only in males from river. Proteins that were not found in males were also detected in female pink salmon samples: protein containing the four-disulphide core domain of whey acidic protein (WAP) 2 and FAM20 C-terminal domain-containing protein (A0A060Y336) in females from all biotopes; uncharacterized protein A0A060Z3K3 and coatomer subunit beta'-like - in females from river; VWFD domain-containing protein - in females from sea.

## 4 Discussion

### 4.1 Cluster analysis proteomic profiles of *O. gorbuscha* blood plasma

Cluster analysis showed that the proteomic profiles of *O. gorbuscha* blood plasma were sex- and within sex the characteristics were environment-specific (different biotopes). These are primarily sex differences in the reproductive system, energy expenditure strategies, behavioral patterns, etc (Makiguchi et al., 2017; Esteve, 2005). It is known that sex had a significant modulating effect on the degree of physiological change (Hruska et al., 2010) while the maturation success and time of spawning tightly associated with location inhabited by fish (Wertheimer, 1984). A clear separation of samples by sex was observed in the present study, demonstrating differences between males and females that are most pronounced during spawning. Males from the marine and riverine biotopes formed two separate clusters, while males from the estuary overlapped with others. Females (except for one sample) collected from the river and estuary showed two separate clusters, and fish from the marine biotope overlapped with others. In males, the protein content changes smoothly with the transition from sea-estuary-river, while in females the most pronounced changes are found with the transition from estuary to river. It is known that pink salmon rich finale maturation in freshwater, the low-salinity in the estuary reduced the destructive effects on maturation of fish in seawater (Wertheimer, 1984). For example, females of sockeye salmon *Oncorhynchus nerka* Walbaum, 1792 exhibited a significant change for gross somatic energy, osmolality, and plasma concentrations of Cl^−^, Na^+^, cortisol, testosterone, 11-ketotestosterone, 17,20β-progesterone, and estradiol (Hruska et al., 2010). The detected differences may allow to identify the most critical changes in the environmental conditions that pink salmon undergo during their spawning migration.

### 4.2 Comparative analysis of plasma protein profiles of *O. gorbuscha* females from different biotopes—marine, estuarine and riverine

During spawning migration, the level of vitellogenin (Vtg) in the blood of females increased. It is significantly higher in females from the river compared to females from the estuary. Vtg is involved in vitellogenesis, an important step of reproduction that involves the synthesis and accumulation of yolk in developing oocytes during oogenesis. Vtg is a phospholipid glycoprotein and a precursor of lipoproteins and phosphoproteins, the major protein components of egg yolk in bony fish, and it is usually found only in the blood of females (Copeland et al., 1986; Marioli and Zarkadis, 2008). This protein is synthesized in the liver of females under the control of oestrogen and is transported by the bloodstream to the ovaries, where it selectively penetrates the growing oocyte by endocytosis (Hara et al., 1983; Tyler et al., 1988; Nuñez Rodriguez et al., 1996; Fujita et al., 2004; Lafont et al., 2016). Once inside the oocyte, vitellogenin undergoes proteolysis and constitutes an important nutrient reserve for developing embryos and larvae (Wallace, 1985; Mommsen and Walsh, 1988). Increased levels of von Willebrand factor (VWF) domain D protein were also found in the blood of females from river compared to females from the estuary. Von Willebrand factor (VWF) is a blood glycoprotein involved in haemostasis, particularly platelet adhesion. Interestingly, VWF acts only as a multimer, and the D domain of this protein is a conserved structural component of vitellogenin in many vertebrates and invertebrates (crustaceans and insects) (Wu et al., 2018; Faiz et al., 2019). The D domain has the function of binding oocyte membrane receptors to vitellogenin, resulting in vitellogenin uptake by oocytes (Opresko and Wiley, 1987). Increased levels of several vitellogenin isoforms, choriogenin, and protein containing VWFD domain in the blood of female pink salmon during migration from sea to river indicated activation of oocyte development processes, which is an integral process of fish preparation for spawning.

Fatty acid-binding proteins (FABPs) are cytoplasmic proteins belonging to a conserved multigenic family that are involved in lipid uptake and transport, regulating lipid metabolism and cellular defence. Various FABP isoforms are expressed in most tissues all vertebrates. Cardiac FABP (H-) and epidermal FABP (E-) are the most abundant. H-FABP is expressed primarily in heart and skeletal muscle but is also present in kidney, lung, mammary tissue, placenta, testis, stomach, and brain. E-FABP is widely distributed in skin, lung, heart, skeletal muscle, kidney, testis, and adipose tissue. The differential pattern of tissue expression and the variation in FABP ligand specificity indicate that FABPs have unique functions under different conditions (Kim, 2006). FABP1, FABP2, FABP3, FABP6, FABP7, FABP10, and FABP11 have been identified in bony fish genomes, with FABP11 being unique to this group. In some fish species, fat deposition can occur in the liver, muscle, and mesentery, but not in adipocytes, which is explained by the absence of the FABP4 gene (AFABP, adipocytes) (Zhang et al., 2020). In the vertebrate ovary, different FABPs have diverse functions and act through various, multidirectional metabolic pathways, such as fatty acid uptake, intracellular transport and utilization processes accompanying folliculogenesis, atresia and hormone synthesis. Increased expression of Fabp11 transcripts was detected in somatic cells of atresic follicles in the Senegalese sole *Solea senegalensis* Kaup, 1858. According to the authors, this indicated the possible importance of lipid metabolism and the involvement of Fabp11 and fatty acids in follicular atresia (Agulleiro et al., 2007). The blood plasma of female pink salmon from the river compared to those from the estuary showed increased levels of cardiac-type FABP, which probably participates in fatty acid metabolism. Our finding supports the statements about the active oocytes development and maturation of pink salmon during migration and finalization of the process in freshwater location. As known these processes undergo along with yolk formation and the organization of the thick membrane and complex lamellar structure of the demersal eggs (Stehr and Hawkes, 1979).

In our previous study (Nefedova et al., 2018; Murzina et al., 2019) we have shown that the metabolically mature pink salmon eggs were characterized by high levels of lipids supply of structural and nutritional components and satisfy embryo demands during development. More, the viability of eggs and further successful development of embryo depends on the specific fatty acid composition and mostly on the amount and ratio of essential fatty acids—eicosapentaenoic and docosahexaenoic fatty acids, and the amount of monounsaturated fatty acids. It was discussed (Murzina et al., 2019) the prelarvae retain a high content of dominant oleic 18:1ω-9 and docosahexaenoic 22:6ω-3 FAs, which are functionally significant as an energy unit and a plastic reserve during the further development of pink salmon larvae. Two proteolytic enzymes were identified in the blood plasma of pink salmon females: serine endopeptidase (peptidase S1 domain-containing protein) and metallo-endopeptidase (leukocyte cell-derived chemotaxin 2 (LECT2)). The levels of these peptidases were increased in females from the estuary in comparison to those from the marine and riverine biotopes. The S1 peptidase family belongs to the group of serine endopeptidases. Leukocyte chemotaxin 2 is a multifunctional protein involved in chemotaxis, liver regeneration, immune response, and bone growth (Segawa et al., 2001; Okumura et al., 2008; Wei et al., 2011; Lu et al., 2013a; Lu et al., 2013b; Slowik and Apte, 2017; Xu et al., 2019). LECT2 has been identified in several organisms, including fish (Kokkinos et al., 2005). The most prominent LECT2 is due to its potential role in fish inflammatory response during pathogenic infection (Shi et al., 2012). Protease enzymes are involved in various aspects of male and female reproductive processes, including gametogenesis and maturation, fertilization, post-fertilization events, and mating behaviour (Kiyozumi and Ikawa, 2022). In the muscles of wild sockeye salmon, changes in metabolic processes during migration to the spawning site in the river have been observed. In particular, an induction of proteolytic reactions was shown, which the authors attribute to increased protein metabolism at the starvation period (Miller et al., 2009). However, in our case, since there is no further increase in proteases in females from the river, these changes are associated with a compensatory response of the fish to stress during salinity changes. While it is important to consider that proteases known as multifunctional enzymes involved in many metabolic reactions depend on the physiological state of an organism.

For females from the river, the coatomer subunit beta'-like, fructose bisphosphatase, liver FABP 10-A basic, and 4-hydroxyphenylpyruvate dioxygenase were specific, i.e., they were not identified in females from sea and estuary. Coatomer is a seven-subunit cytoplasmic protein complex involved in the intracellular transport of vesicles from the Golgi apparatus to the endoplasmic reticulum and back (Cosson and Letourneur, 1997; Hahn et al., 2000; Moelleken et al., 2007). Fructose 1,6-bisphosphatase is implicated in glucose synthesis (gluconeogenesis). It catalyses the conversion of fructose 1,6-bisphosphate to fructose 6-phosphate in fish liver (Hemre et al., 2002). 4-Hydroxyphenylpyruvate dioxygenase (HPPD) is a Fe(II)-containing non-heme oxygenase involved in tyrosine catabolism, the products of which are used in energy metabolism (Moran, 2005; Salamanca et al., 2020). These proteins are involved in intracellular transport and energy supply processes, which are probably aimed at maintaining the organism’s functions in this final and resource-consuming stage of the fish’s life cycle.

### 4.3 Comparative analysis of plasma protein profiles of *O. gorbuscha* males from different biotopes—marine, estuarine and riverine

The levels of several isoforms of fibrinogen (fibrinogen alpha, beta and gamma chains, fibrinopeptide A), FEL-like protein, and vitellogenin domain protein varied in fish blood during the transition of males from sea to river. Fibrinogen is among the major plasma proteins involved in platelet aggregation, blood clotting, and immune response in fish (Podolnikova et al., 2003; Li et al., 2022). Plasma fibrinogen levels in fish can be influenced by exogenous factors, e.g., lack of food, reduced feeding intensity or environmental alterations (water temperature, season), and by endogenous processes associated with resource consumption for gonadal development (Siddiqu, 1977). As a result of stress, an increase in plasma fibrinogen levels is usually observed in fish (Raposo de Magalhães et al., 2020; Berezina and Fomina, 2021). In our study, the decrease in these protein levels was probably the result of the influence of several such factors, which male pink salmon are exposed to during spawning migration from sea to river. One such factor may be the depletion of fat reserves and increased consumption of protein for energy.

Increased levels of a protein containing the vitellogenin domain in the plasma of males have been observed during migration from sea to river. Due to the lack of stimulation of the liver to synthesize vitellogenin, it is absent or circulates in very small amounts in the blood of males (Mewes et al., 2002). The role of this protein in males is currently unclear. It is known that vitellogenin is used as a marker for environmental contamination by hormone-like substances (Matozzo et al., 2008). Lectins are receptors capable of specifically binding to pathogen surface carbohydrates and promoting their rapid removal through opsonization and phagocytosis (Weis and Drickamer, 1996; Saito et al., 1997; Weis et al., 1998; Janeway and Medzhitov, 2002; Nauta et al., 2004; Yang et al., 2008). There was a decrease in FEL content in the blood plasma of males during migration. Lectins belonging to the FEL group have been identified in the eggs of several bony fish species (Shiina et al., 2002; Wang et al., 2016). FELs are involved in various immune responses in fish (Zhang et al., 2020). The weakening of immune function in mature migrating salmon is probably not due to pathogenic infection or cytokine-mediated immune suppression but rather to a decrease in energy stores or hormonal changes in fish during spawning (Dolan et al., 2016). Quantitative strategy of the reproductive success is characterized by males focusing on mating with as many females as possible (De Gaudemar, 1998; Quinn, 2005). To maintain this strategy, a strong innate immune response is carried out by antibody-independent mechanisms including complement activation and antimicrobial peptides in males (Dolan et al., 2016). Along with this higher spleen index in males helps to sustain blood filtering leading to increase capacity for both antibody and innate immune responses necessary for survival. Мannose-specific lectin-like protein (MBL) was identified only in males from the estuary and river. MBL is a plasma protein involved in the lectin pathway of complement activation, one of the humoral mechanisms of innate immunity. MBL is activated by binding to mannose residues and several other carbohydrates that are part of the cell wall of a number of pathogens. This reaction activates MBL-associated serine proteases that cleave and thereby activate complement components involved in pathogen lysis (Nakao et al., 2006; Dang et al., 2018). In addition, the enzyme peptidyl-prolyl cis-trans isomerase (PPIase) was identified only in the plasma of males collected from the sea. PPIases are involved in the folding of synthesized proteins by catalysing the cis/trans isomerization of proline-peptide bonds. Peptidyl-prolyl isomerases have been implicated in immune function, cell cycle control, and pathogenesis (Kromina et al., 2008; Rostam et al., 2015). It is likely that the variability in the levels of proteins involved in immune responses in the blood plasma of males is related to changes in hormonal status during the migration to the spawning site (Harris and Bird, 2000; Szwejser et al., 2017; Chaves-Pozo et al., 2018; Valero et al., 2018).

### 4.4 Comparative proteomic analysis of male and female *O. gorbuscha* plasma

Blood plasma protein profiles of females and males collected from three biotopes (marine, estuarine and riverine) consequentially passed by spawners during spawning migration were quantitatively and qualitatively different. The differentially expressed plasma proteins of pink salmon, according to the annotation of the Gene Ontology terms (Ashburner et al., 2000), included mainly proteins involved in lipid transport [vitellogenin As, uncharacterized protein (vitellogenin), protein containing the D domain of VWF, uncharacterized protein (apolipoprotein A-IV)], proteolysis (aminopeptidase and protein containing the peptidase domain S1), blood coagulation, platelet activation, and protein polymerization (fibrinogen β chain, fibrinogen gamma chain). Sex differences in the blood plasma proteome of pink salmon are probably caused by the specificity of reproductive function and by the energy metabolism strategies. *Salmo salar* L. is characterized by sex differences in the strategies of energy metabolism. For example, energy expenditure during spawning is similar in female and male salmon, but the energy in females is mainly directed to gonadal development, whereas energy in males is directed to somatic systems (Jonsson et al., 1991; Penney and Moffitt, 2014). Findings from the our current studyfor the lipid profile analysis of certain tissues of the studied female and male fish showed sex specificity in certain polar and neutral lipid classes that are selectively used in metabolism. Accumulated lipids are likely to be the primary source of energy during the upstream migration and in the early stages of gonad development in males and females Oncorhynchus species (Hendry et al., 2000; Penney and Moffitt, 2014). Protein is metabolized during spawning after fat stores are depleted (Hendry and Berg, 1999; Kiessling et al., 2004). Protein content in mature male steelhead salmon *Oncorhynchus mykiss* Walbaum, 1792 was reported to be consistently higher than in females (Penney and Moffitt, 2014). As a result, the consumption of lipids, which are lower in males than in females, is observed first, and then proteins become a source of energy. This is consistent with the observed decrease in fibrinogen in the blood of males and the lower levels of proteins involved in lipid metabolism compared to females.

Unique (sex-specific) proteins were identified only in male and female pink salmon. Proteins such as vitronectin b, a protein containing the TGF_BETA_2 domain, ceramidase, an uncharacterized protein A0A060Z7F0, and a CD59-like protein were identified only in the blood of males. Ceramidase is a lysosomal enzyme that cleaves fatty acids from ceramide to form sphingosine (Parveen et al., 2019). Ceramidase is implicated in maintaining a balance between ceramide and sphingosine-1-phosphate levels, which in part determines cell ‘fate’ as ceramide is involved in stress-related cellular responses and apoptosis, and sphingosine-1-phosphate stimulates cell survival and proliferation and tissue regeneration.

Vitronectin is a glycoprotein and one of the major protein components of blood plasma. This protein participates in fibrinolysis, mediates cell adhesion and migration, inhibits the membrane-attacking cytolytic complex of the complement system, and binds some serpins (Marioli and Zarkadis, 2008). TGF-b is an evolutionarily conserved growth factor family cytokine involved in a variety of biological processes, including cell growth and differentiation, extracellular matrix regulation, and immune response (Wang et al., 2019). CD59 is a complement regulatory protein that is an inhibitor of the formation of the complement membrane attack complex (Sui et al., 2016). Interestingly, vitronectin, TGF-b and CD59-like protein are involved in different pathways of innate immune response in fish. (Rebl and Goldammer, 2018). It is known that fish undergo sex-specific changes in their immune systems during spawning, which are modulated by hormones (Harris and Bird, 2000; Yada and Nakanishi, 2002; Szwejser et al., 2017; Chaves-Pozo et al., 2018; Valero et al., 2018; Campbell et al., 2021). Recently it was shown that sockeye salmon are capable of inducing B cell responses during their spawning migration and male performed significant responses compared to females (Smith and Zwollo, 2020). B cell activation by spawners provide additional protection against pathogens not encountered as juveniles. More, authors observed the ability to retain B lymphopoiesis by fish during the early stages of freshwater migration that could be an indicator for pre-spawning survival further to the moving to spawning grounds. Smith and Zwollo. (2020) discreetly discuss that mature female of sockeye salmon were are more immunocompromised compared to males, due to higher cortisol, testosterone and/or 17β-estradiol levels, concluding to suppress antibody responses during the final stages of the salmon’s life cycle.

A protein containing the four-disulphide core domain of whey acidic protein (WAP) 2, FAM20 C-terminal domain-containing protein (A0A060Y336) uncharacterized protein A0A060Z3K3, VWFD domain-containing protein, and coatomer subunit beta'-like were found only in female blood plasma. WAP is a milk serum component of some mammals. However, the large four-disulphide domain of WAP is found not only in this protein but also in many other proteins involved in various biological processes, among which immune responses are predominant (Zhang et al., 2020). Role of WAP in the immune system of fish remains unclear. The protein containing the VWFD domain is implicated in vitellogenin transport into oocytes, and the beta-like coatomer subunit participates in intracellular transport processes as described above. FAM20 C-terminal domain-containing protein is a member of the FAM20 family of secreted proteins with potential roles in regulating differentiation and function of hematopoietic and other tissues (Nalbant et al., 2005). It is not clear how this protein specifically functions in fish.

Thus, this study revealed changes in the blood plasma proteome compositions of female and male pink salmon *O. gorbuscha* during spawning migration. These changes primarily affect proteins involved in reproductive system development, lipid transport, and energy production in females, and proteins involved in blood coagulation, immune response, and reproductive processes in males. Among the differentially expressed sex-specific proteins were pink salmon blood plasma proteins implicated in immune response, cell growth and differentiation, and lipid transport processes. The results are of fundamental and practical importance because they add to existing knowledge of biochemical adaptations to spawning migration in pink salmon, which is representative of commercial migratory fish species.

## Data Availability

The datasets presented in this study can be found in online repositories. The names of the repository/repositories and accession number(s) can be found below: https://www.ebi.ac.uk/pride/; PXD042278.
