# Supplementary material for: Proteins journey—from marine to freshwater ecosystem: blood plasma proteomic profiles of pink salmon Oncorhynchus gorbuscha Walbaum, 1792 during spawning migration
Source: Front Physiol. 2023 Jun 13;14:1216119. doi: 10.3389/fphys.2023.1216119 (PMC10293649; doi:10.3389/fphys.2023.1216119)
Supplement: Supplementary file 1 [file Table2.DOCX]

Supplementary Material

Proteins journey - from marine to freshwater ecosystem: blood plasma proteomic profiles of pink salmon *Oncorhynchus gorbuscha* Walbaum, 1792 during spawning migration

Albina Kochneva*, Denis Efremov, Svetlana A. Murzina

*** Correspondence:** Corresponding Author: [kochnevaalbina@gmail.com](mailto:kochnevaalbina@gmail.com)

**Supplementary Tables S1-S4**

**Table S1** The raw LFQ-intensity values of the identified proteins of male and female *O. gorbuscha* from different biotopes (marine, estuarine and riverine).

**Table S2** Filtered and logarithmized LFQ-intensity values of the identified proteins of male and female *O. gorbuscha* from different biotopes (marine, estuarine and riverine).

**Table S3** Filtered, logarithmic, and imputed LFQ-intensity values of the identified proteins of male and female *O. gorbuscha* from different biotopes (marine, estuarine and riverine).

**Table S4** Differentially expressed plasma proteins of *O. gorbuscha* male and female from different biotopes (marine, estuarine and riverine)
